# Key challenges of health care workers in implementing the integrated management of childhood illnesses (IMCI) program: a scoping review

**DOI:** 10.1080/16549716.2020.1732669

**Published:** 2020-03-02

**Authors:** Mark Donald Reñosa, Sarah Dalglish, Kate Bärnighausen, Shannon McMahon

**Affiliations:** a Heidelberg Institute of Global Health, Ruprecht-Karls-Universität Heidelberg, Heidelberg, Germany; b Department of Epidemiology and Biostatistics, Research Institute for Tropical Medicine - Department of Health, Manila, Philippines; c Johns Hopkins Bloomberg School of Public Health, Baltimore, MD, USA; d School of Public Health, University of Witwatersrand, Johannesburg, South Africa

**Keywords:** Child mortality, child health, childhood illness, program design and implementation challenges

## Abstract

**Background**: Several evaluative studies demonstrate that a well-coordinated Integrated Management of Childhood Illnesses (IMCI) program can reduce child mortality. However, there is dearth of information on how frontline providers perceive IMCI and how, in their view, the program is implemented and how it could be refined and revitalized.

**Purpose**: To determine the key challenges affecting IMCI implementation from the perspective of health care workers (HCWs) in primary health care facilities.

**Methods**: A scoping review based on the five-step framework of Arskey and O’Malley was utilized to identify key challenges faced by HCWs implementing the IMCI program in primary health care facilities. A comprehensive search of peer-reviewed literature through PubMed, ScienceDirect, EBSCOhost and Google Scholar was conducted. A total of 1,475 publications were screened for eligibility and 41 publications identified for full-text evaluation. Twenty-four (24) published articles met our inclusion criteria, and were investigated to tease out common themes related to challenges of HCWs in terms of implementing the IMCI program.

**Results**: Four key challenges emerged from our analysis: 1) Insufficient financial resources to fund program activities, 2) Lack of training, mentoring and supervision from the tertiary level, 3) Length of time required for effective and meaningful IMCI consultations conflicts with competing demands and 4) Lack of planning and coordination between policy makers and implementers resulting in ambiguity of roles and accountability. Although the IMCI program can provide substantial benefits, more information is still needed regarding implementation processes and acceptability in primary health care settings.

**Conclusion**: Recognizing and understanding insights of those enacting health programs such as IMCI can spark meaningful strategic recommendations to improve IMCI program effectiveness. This review suggests four domains that merit consideration in the context of efforts to scale and expand IMCI programs.

## Background

The World Health Organization (WHO), the United Nations Children’s Fund (UNICEF) and other partners developed the Integrated Management of Childhood Illnesses (IMCI) strategy in the mid-1990s to address child mortality and improve health care workers’ (HCWs) ability to diagnose, classify, and manage childhood illnesses [1]. IMCI, a case-management strategy for common childhood illnesses, is designed primarily for non-physician clinicians working in resource-poor settings. IMCI guidelines are framed as a series of simple questions in a step-by-step algorithm, meant to help HCWs classify illnesses based on presenting signs and symptoms, severity and danger signs [2–5]. When used correctly, these guidelines have been shown to improve quality of care, and reduce the cost of treatment [6–8]. Since its inception, two key goals of the IMCI program have been to strengthen the capacity of HCWs to manage childhood illnesses, and to increase mothers’ abilities to recognize danger signs [5,9,10]. Despite the successes of the IMCI program, few countries have achieved full expansion of IMCI implementation, and coverage remains problematic even after 20 years of IMCI implementation [8,11].

While there have been a number of systematic and analytic reviews conducted on IMCI, most emphasize the early stages of implementation and how IMCI contributes to reductions in childhood mortality and its effect on health systems [7,12]. Hansoti and colleagues [13] have provided information on the reliability and validity of the IMCI tool in low- and middle-income countries (LMICs). Still other reviews have evaluated IMCI trainings in terms of effectiveness of a shortened training [14], and whether trainings improve HCWs performance [15,16]. UNICEF [11] published a working paper highlighting diverse scientific and programmatic evidence on IMCI from its establishment to the present. Poor families were viewed as greatly benefitting from IMCI because it could, in theory, increase equitable access to basic childcare services in revitalized and enabled the primary health care (PHC)-facilities [3,17]. However, evidence suggests implementation has suffered from larger structural problems and weakness in IMCI program execution by global and in-country partners [8,11,12]. Jacobs and Merson [18] note the failures of IMCI to provide continuous care between the home, first level PHC-facilities and referring health facilities. Generally speaking, IMCI’s impact on reducing inequity is difficult to assess given the varied extent of implementation [19].

Although the existing body of evidence provides analysis of the impact of IMCI, little information exists regarding how the strategy can best be delivered in PHC-facilities from the perspective of HCWs [7]. There is relatively less evidence in terms of how providers, who are the key actors in implementation, perceive the IMCI program in terms of quality, relevance, acceptability and sustainability.

To address this gap, we conducted a scoping review focusing on the experiences and perspectives of HCWs implementing IMCI in PHC-facilities in LMICs. This review aimed to provide a synthesis of the data and to lay the groundwork for health professionals, public health practitioners and policy makers to draw on perspectives of those at the frontlines of IMCI delivery when determining whether or how to refine program implementation.

## Methods

The scoping review approach was based on the framework developed by Arksey and O’Malley [20], which outlines an iterative and reflexive way of understanding the extent and range of the scientific literature on a given topic. The five-step framework entails: 1) identifying the research question, 2) identifying relevant studies, 3) selecting studies, 4) charting the data, and 5) collating, summarizing and reporting the results.

### Identifying the research question

A brainstorming session was conducted to develop potential scoping questions focused around IMCI program implementation. The preliminary question was sent to members of the study team to evaluate its relevance and applicability. After discussions, the scoping question was refined to the following: determine the key challenges affecting IMCI implementation from the perspective of HCWs in PHC-facilities.

Following the finalization of the scoping question, core concepts were discussed and a set of keywords identified and narrowed down after checking Medical Subject Headings (MeSH) browser, including Non-MESH terms. Boolean operators were used to exhaust the variations of terms scoped. The final search strings and electronic databases searched are shown in Table 1.

**Table 1.** Electronic databases and search strings of the IMCI scoping review, 2018–2019

Electronic database	Search String
PubMed EBSCOhost ScienceDirect Google Scholar	(Search (((((((((((Integrated Management of Childhood Illnesses) OR Integrated Management of Childhood Illness) OR Integrated Management of Childhood Illnesses Program) OR IMCI) OR IMCI Program) AND Health care providers) OR Health care workers) OR Primary health care workers) OR Doctor) OR Nurse) OR Midwife) AND Challenges in Implementation Filters: Full text; Humans; English))

### Identifying relevant studies

The lead author performed all searches from 27 December 2018–15 January 2019. Three electronic databases were used: PubMed, ScienceDirect and EBSCOhost, to identify peer-reviewed journals relating to the scope of review. Google Scholar was also used to search for publications in the grey literature. Only studies in English and only scientific papers from 2005 to 2018 were included. The majority of LMICs introduced the program by 1999–2004 and initial analytic reviews and a multi-country evaluation study were conducted in 2002–2005 [21]. The 2005–2018 window was therefore selected to capture countries in the implementation and expansion phases discussed by the WHO Guidelines on IMCI Program [8]. A full list of inclusion and exclusion criteria are outlined in Table 2.

**Table 2.** Inclusion and exclusion criteria of the IMCI scoping review, 2018–2019

CRITERION	INCLUSION	EXCLUSION
*Types of Participants*		
Population and sample	Health care providers (Doctors, Nurses and Midwives) in low- and middle-income countries	
*Concept & Context*		
Literature focus	Original research and/or scientific papers related to experiences of implementing the IMCI program. Key challenges influencing the poor uptake of IMCI program.	Studies focuses on hospital-based implementation of IMCI program Studies focuses on Integrated Community Case Management (iCCM) Strategy (only focuses on 3 diseases: malaria, pneumonia & diarrhea)
Time Period	2005 to 2018	
Language	English	
Type of article	Original research and/or scientific papers published in a peer-reviewed journal *(with retrievable full-text)* Studies with ethics approval	Articles that are editorials, commentaries, research summaries, discussion papers, policy and strategic reviews or personal viewpoints. Articles that are abstract only and/or no retrievable full-text

### Study selection and charting the data

The lead author listed all details, assessed and read the abstracts and evaluated whether they met the inclusion criteria, and noted themes emerging from analysis using Microsoft Excel. A data extraction spreadsheet was developed to chart the information in the literature, adapted from The Joanna Briggs Institute Reviewers’ Manual [22]. Details were extracted regarding publication information, study design, methodology, sample population (if applicable), and all relevant information pertaining to the scoping questions.

### Collating and summarizing the results

We used descriptive statistics to summarize all included studies. Counts and proportions were used to describe all other information gathered such as specific research design, publication details, and locale of the study. We also conducted a content analysis to summarize key concepts from the studies and relating to the study scope of interest [23]. Each text pertaining to the experiences of the HCWs implementing IMCI program was coded or broken down into categories on a multi-level: words, word sense, phrase, sentence or theme. All included studies were read and re-read for content on the HCWs’ experiences and professional challenges. The lead author, working on printed copies of the studies with markers and highlighters, conducted the coding manually. Relevant statements were highlighted, with notes taken on themes emerging from our analysis. These notes helped to progress the substance of meanings and depth of understanding on the HCWs experience. All relevant data from the final included studies were extracted into the data extraction spreadsheet in Microsoft Excel. Coded data extracts for each theme were reviewed by the members of the study team (see Supplementary Table 1) to ensure patterns were clearly understood and defined. Themes were organized and reorganized through collaborative dialogues via Skype calls and email exchanges to arrive at consensus. We structure our results around common themes, to show the extent and range of themes within included studies. Initial analysis emphasizes various levels of implementation challenges. Later analysis led to the development of a four domains framework, reflecting the connections and relationship between each theme.

## Results

**Figure 1.**
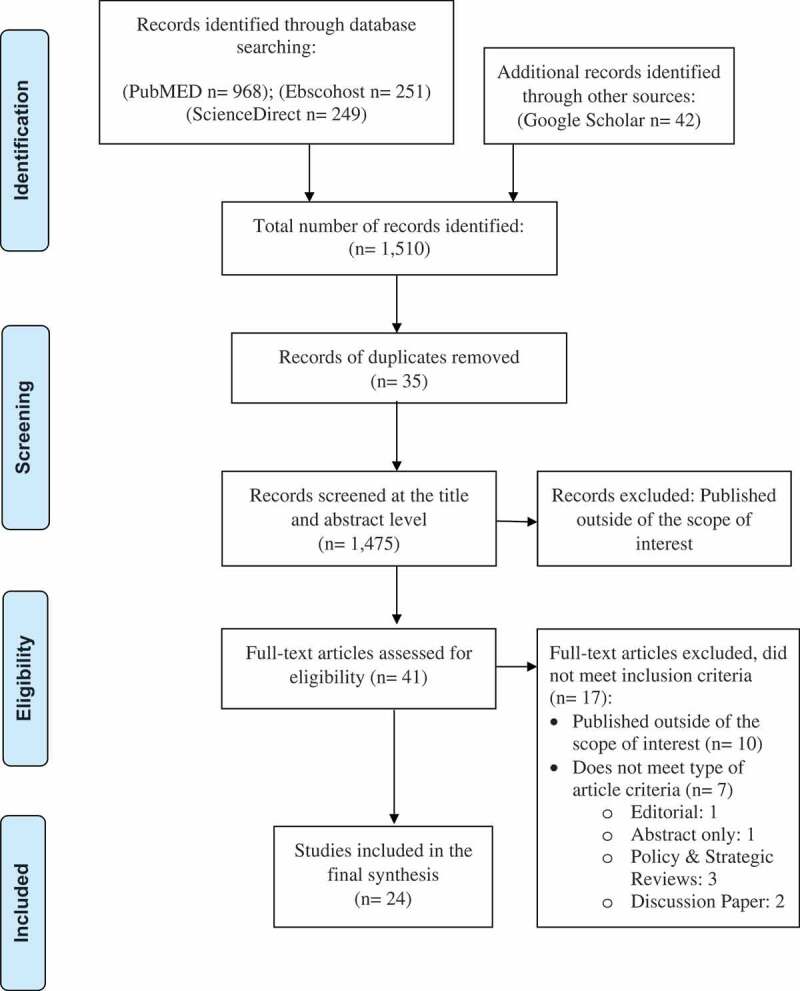
PRISMA diagram of articles identified and selected

A total of 1,475 publications were screened and assessed for eligibility based on inclusion and exclusion criteria. Figure 1 outlines this process. A total of 1,434 publications were screened out on initial assessment of the title and abstract; 41 publications were read full-text. Of these, 17 publications did not meet the inclusion criteria because their content was outside the scope of interest and/or they were not of the appropriate article type.

### Characteristics of the included studies

This scoping review yielded 24 studies (see summary in Supplemental Table 1). Among these studies, 11 utilized quantitative designs (cross-sectional surveys, exploratory and observational surveys); eight were mixed-method designs, and the rest used a qualitative design. Three papers utilized a multi-country evaluation survey design. A majority of included studies were conducted in the WHO African region (65%), followed by the Eastern Mediterranean region (15%) and South-East Asia (10%); the remaining studies were conducted in the Americas, Western Pacific and European regions. The reviewed studies differed in study design, number of participants and length of the study. All included studies were published as research articles in peer-reviewed journals, most frequently in Health Policy and Planning (16.7%), BMC Health Services Research (8.3%), BMC Public Health (8.3%), PLOS One (8.3%) and Global Health Action (8.3%).

### Key findings

**Figure 2.**
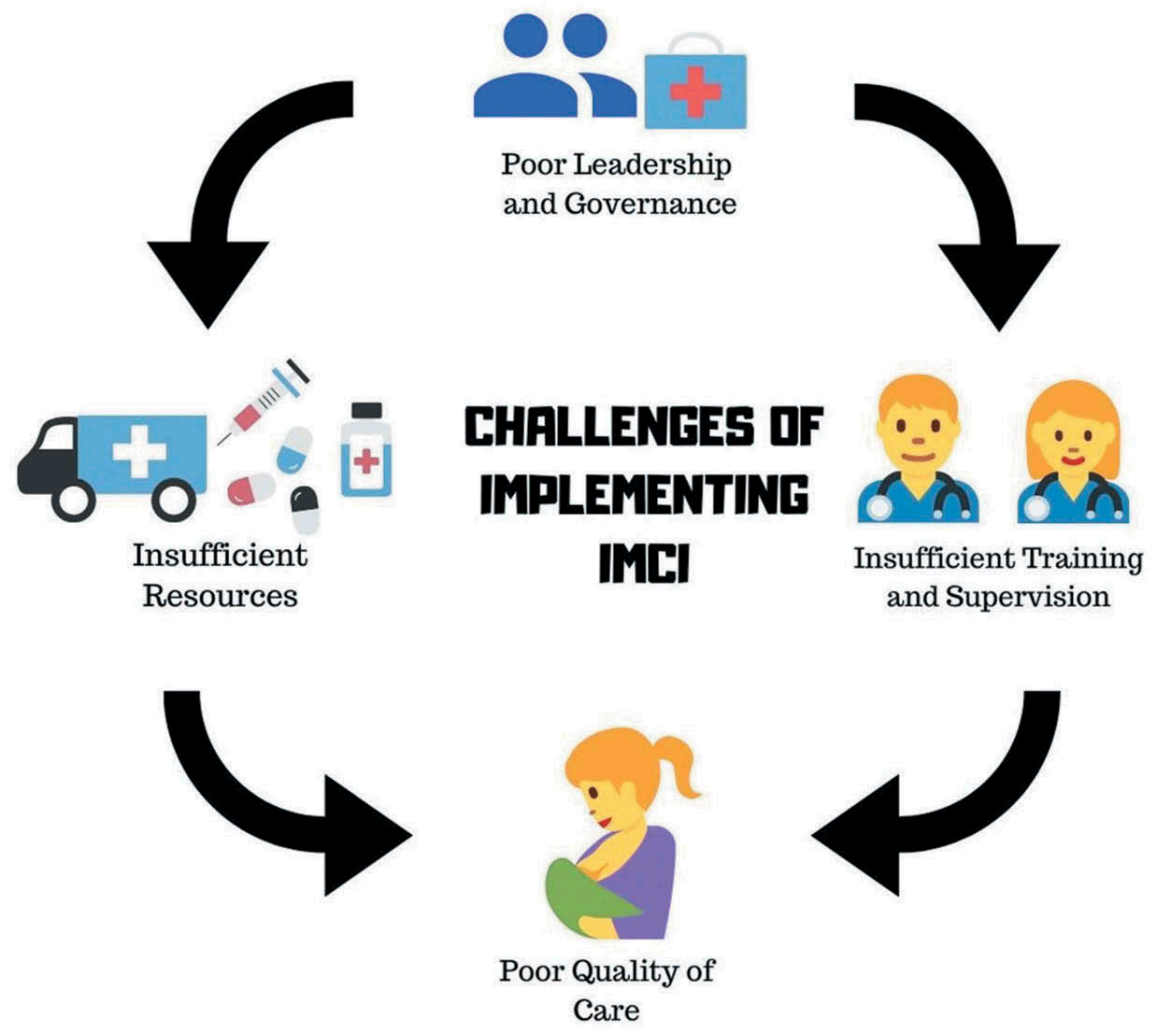
Interplays of four main domains in IMCI implementation. *Icons credit*: courtesy of www.canva.com

Table 3 presents the key findings of the included studies, organized by theme. The findings identified the existing barriers reported in the implementation of the IMCI program, which limited the accomplishment of its program objectives, from the point of view of HCWs in PHC-facilities. Bottlenecks and challenges across four domains – (1) leadership and governance; (2) resources; (3) training, mentoring and supervision; and (4) quality of care – impede the optimal performance of the HCWs in delivery of IMCI services by causing staff turnover, demotivation and burnout. Figure 2 shows the interplays of the four main domains identified by this review. It highlights the negative effect of poor leadership and governance, lack of resources and inadequate training, all leading to poor performance of HCWs – and compromising care – in PHC-facilities.

**Table 3.** Key findings of the included studies in the scoping review, 2018–2019

THEMES	KEY CHALLENGES	EFFECT ON HCW DELIVERING IMCI PROGRAM
Leadership and Governance	Poor dynamics (lack of planning and coordination between policy makers to HCW implementers) and ineffective decentralization. Other child health programs (such as Expanded Program of Immunization, Tuberculosis, Nutrition and Malaria control) were prioritized and not harmonized to the IMCI program. Donors shifted their interests to ICCM (Integrated community case management program) and neglected the IMCI program. Absence of IMCI institutionalization (i.e. no specific budget allocation) at district and PHC-levels affected its prioritization and rollout.	HCWs had unclear roles and uncertainty on the expected tasks for IMCI program implementation. HCWs had difficulty synchronizing some tasks in PHC-facilities. HCWs lack support to continue the IMCI program. HCWs felt that they were losing time due to administrative burdens and required reports.
Resources for IMCI Implementation	Inadequate supply of essential medicines, IMCI wall charts and booklets, lack of basic equipment and transport for referrals. Shortage of trained HCWs at PHC-facilities. Lack of enabling and supportive health facility structures.	HCWs were dissatisfied with the working conditions because they lacked adequate supplies to do their job. Trained HCWs were burned-out because of too many tasks to perform. HCWs cannot render some vital IMCI services, such as counseling of caretakers.
Training, Mentoring and Supervision	Long duration and high cost of IMCI training, insufficient follow-up after training, and unavailability of refresher courses. Low number of skilled IMCI training facilitators and lack of appropriate training sites. No standard IMCI-specific supervision and lack of motivation of supervisors. Lack of funding for follow-up after training and inadequate job aids.	HCWs needed to leave their workplace in long days, creating problems of lack of personnel to manage the PHC-facilities. HCWs who were not trained on IMCI inhibit scale-up in some LMICs. HCWs compliance to IMCI algorithm was uneven and often led to incorrect classification even after IMCI trainings. HCWs who are meant to do supervision and monitoring cannot perform their duties.
Quality of Care	IMCI child health assessment protocols were not consistent and comprehensive. Length of time needed for IMCI consultations and overall poor working conditions for HCWs	HCWs struggled to understand and consistently implement IMCI leading to misclassification and missed referrals. HCWs often failed to do nutritional assessments such as searching for signs of malnutrition, and providing caretakers with advice on feeding practices was usually omitted.

#### Leadership and governance

Nine studies found that poor leadership dynamics and ineffective decentralization were among the main causes of failures in implementation of IMCI programs. A lack of planning and coordination, and wide communication gaps between various stakeholders, contributed to problems of limited coverage. Pradhan and colleagues [24] expounded the ambiguity of the roles given to the HCW implementers. HCWs in Pakistan were uncertain about expected tasks for IMCI program implementation. An IMCI trained district level implementer said, *‘The ball is thrown in our court after providing IMCI training by MNCH [Maternal and Neonatal Child Health] program. I am helpless at this level … I am helpless …* [24],*’* showing the disconnect between policy makers and implementers. Furthermore, in South Africa, Pandya and colleagues [25] concluded that the lack of clarity of roles and accountability among various stakeholders were critical components of IMCI implementation failures.

Pandya and colleagues [25] highlighted that there is a problem with integrating IMCI into existing health programs, which caused fragmentation of governance structures that influence the IMCI program and other child health programs. Other vertical programs such as expanded program on immunization (EPI) and tuberculosis were often prioritized and not synchronized to the IMCI program. Moreover, the coordinators managing these vertical programs were not trained in IMCI, creating a lack of awareness of the IMCI aspects within these child health programs. In Yemen, Basaleem and Amin [26] also mentioned a lack of harmonization between IMCI and other programs in the health system, including overlapping programs on nutrition, immunization and malaria control, magnifying problems with poor IMCI implementation. HCWs sometimes said they were better off without IMCI due to inflexible rules and a lack of integration with other programs. They felt that they were losing time due to IMCI’s huge administrative burden and required reports.

Meanwhile, Huicho, et al [27] found that one of the major constraints in scaling-up IMCI in Peru stemmed from problems within the health sector policy and strategic management levels. The lack of IMCI institutionalization at ministry or district levels had profound effects on service delivery in PHC-facilities. The lack of political commitment, poor governance, absence of specific budget allocations, and a failure to prioritize child health at the national level were also highlighted. Goga and Muhe [28] also reported that among the challenges in scale-up in six countries was a lack of buy-in from the national stakeholders. Nsabagasani, et al [29] in their study in Uganda also mentioned that donors shifted interests to the Integrated Community Case Management (iCCM) program and abandoned the IMCI program. One Ministry of Health official said: *‘At the moment we do not have money for continuing some of the IMCI activities the way we are supposed to. Donors have shifted to iCCM. We have adopted the IMCI approach as a routine, but we do not have needed resources to sustain some of the core activities including revision of guidelines and refresher training* [29]*’*.

#### Resources

A majority of studies (17 of 24) highlighted issues of inadequate human and material resources. These studies found that some of the IMCI recommendations are not carried out because of insufficient support from the government and stakeholders. Vhuromu & Davhana-Maselesele [30] explored the experiences of HCWs in South Africa, highlighting the lack of human and material resources as one of the reasons that hampered the implementation of IMCI services in Limpopo province. Because of problems with shortages of medication, HCWs said there were instances that they referred patients to nearby hospitals just to get medications, even if it was not otherwise necessary. Physical structures did not support the delivery of IMCI services (for example, the rooms were not big enough to render counseling services). Participants in the study said, *‘There is no open space as the rooms are very small. This fails us to implement the rehydration plan for a dehydrated baby according to the IMCI standards* [30]*’*. Such poor working conditions led HCWs to suffer from ‘burnout’ (such as exhaustion and poor morale), which affected the uptake of the IMCI program.

The basic materials and supplies required to provide care under IMCI were often lacking. In Ethiopia, Seid and Sendo [31] showed that 57% of nurses in four districts reported a lack of essential drugs, IMCI wall charts and chart booklets. Pandya, et al [25] in their study in Gauteng province in South Africa also reported major deficiencies in medical supplies, such as a shortage of growth monitoring equipment, as well as vitamin A and deworming drugs, due to the poor performance of the centralized drug dispensing system. In Yemen, Basaleem & Amin [26] found shortages of mothers’ counseling cards (the IMCI recommendation was to give one to every mother), as well as the unavailability of thermometers and issues with drug supply. Participants in the study said, *‘The last time we received drugs was 6 months back, and most of the commonly prescribed drugs are either finished or about to finish and we will not receive a new supply in the near future* [26].*’* Because of these inadequacies, HCWs were dissatisfied with the working conditions when applying some of the IMCI recommendations.

#### Training, mentoring and supervision

Several studies (14 of 24) highlighted the short duration and cost of IMCI training, as well as poor supervision and lack of follow-up after training, which affected the performance of HCWs. Horwood, et al [4] in their study in Limpopo and Kwazulu-Natal province in South Africa found that the standard 11-day IMCI training was too short to acquire all skills required and that the follow-up after training was insufficient to ensure competency in the conduct of the comprehensive assessments. HCWs’ compliance to IMCI algorithms was uneven and HCWs often made incorrect classifications even after the training. The study found a need to re-evaluate the training materials and methods to ensure that the IMCI program was properly implemented.

Goga and Muhe [28] also concluded in a multi-country survey of 27 countries (representing the six WHO regions) that a majority of countries struggled to conduct follow-ups after IMCI training, mainly due to insufficient funding to cover travel costs, a limited number of trained supervisors, and inadequate job aids for follow-up. Difficulties in achieving adequate reach and coverage of IMCI training were also reported; HCWs who were supposed to implement IMCI needed approximately 11–16 days of training, according to WHO guidelines, but were criticized by national IMCI focal persons or representatives from Ministries of Health for requiring this amount of time. IMCI training was found to be logistically difficult and expensive, as HCWs needed to leave their workplace, thus creating problems of lack of personnel to manage the PHC-facilities during trainings. The cost and time for these trainings, frequent changes in staff and a lack of skilled facilitators and training sites significantly contributed to limited uptake of implementation of the IMCI program. Rowe and colleagues [32] in southeastern Benin also revealed obstacles of supervision at multiple levels of the health system. IMCI supervision checklists were too long, leading to demotivation. Among the root causes of why supervision was not effectively occurring, there was also no standard method of supervision, a lack of committees to coordinate supervision and problems of decentralization.

Seid and Sendo [31] also reported that 89% of trained HCWs were not getting any supervision, and that training was inadequate due to limited mentoring and a lack of refresher courses. HCWs in South Africa and India also shared the same sentiments, namely that support from supervisors was poor and no regular supervision was conducted [30,33]. Huicho, et al [27] found that only 43.5% of trained HCWs in Peru received follow-up visits, and that those supervisory activities were not IMCI-specific and were usually conducted as part of other vertical programs (such as immunization, acute respiratory infections). Facilitators and course directors in a multi-country survey also experienced difficulties integrating IMCI follow-up activities into their routine duties and responsibilities, and facilitators felt that their presence was unwelcome during site visits [28]. Results also highlighted how hierarchical tensions between HCWs and supervisors contributed to demotivated IMCI staff [28]. HCWs viewed supervision as a punitive rather than positive or corrective procedure. One participant said, *‘Participants feel supervisors will police them* [28]*’.*

#### Quality of care

Nearly half of the articles included in the review (10 of 24) found that HCWs’ IMCI assessments were not performed consistently and comprehensively, and that clinical activities not related directly to the presenting complaint were frequently omitted. Such incomplete case assessments could lead to misclassification and missed referrals. For example, in Ethiopia, Gerensea and colleagues [34] documented that out of 384 cases, 37.2% were classified incorrectly and 57.3% were wrongly treated. Despite nationwide training and expansion of the program, in their study of 4 sub-Saharan African countries (Namibia, Kenya, Tanzania and Uganda), Krüger and colleagues [35] also reported that HCWs failed to recognize IMCI danger signs and primary symptoms, which are crucial for the succeeding steps in the IMCI algorithm. Moreover, in South Africa nutritional assessments such as checking for signs of malnutrition were not conducted, and providing advice on feeding practices was usually omitted [4]. Thus, HCWs often failed to promote breastfeeding and provide counseling about complementary feeding.

There was notably poor adherence to IMCI case management procedures regarding the prescription of antibiotics [36]. Naimoli and colleagues (2006) showed that IMCI-trained HCWs do not always follow the guidelines. Specifically, physicians with higher levels of pre-service training tended to prescribe more antibiotics to children, since they felt that the guidelines were only suggestions and not binding guidance.

Further, HCWs faced problems with long IMCI consultations, given the shortage of manpower in their clinics, resulting in longer waiting hours for patients and making it impossible to implement it properly [31,37,38]. HCWs reported needing 20 minutes to an hour for a single IMCI consultation, causing longer patient waiting times, or requiring HCWs to skip steps in the IMCI algorithm due to time constraints [25,39]. As one participant said, *‘The problem is when there are a lot of people in the clinic, there is no time for you to go through all those things for the child. You will be working fast fast fast to push the queue. Sometimes, sisters (nurses) will just be looking at conditions that the child has been brought with and then they leave the rest and you find that the child has not received immunizations and stuff like that* [25]*’*. Further, the IMCI-trained HCWs were also required to provide other health care services, which limited their availability, creating a cycle of insufficient staffing [38].

## Discussion

This scoping review examined a range of factors that impede the uptake and implementation of IMCI from the perspective of HCWs, providing further detail to the implementation challenges discussed in Boschi-Pinto and colleagues’ study [40], which provided a high-level and global view of IMCI implementation. While earlier reviews such as Bryce’s [21] highlighted the positive role and contribution of the IMCI program in strengthening health systems, more recent literature highlights the uneven nature of IMCI implementation across countries. Our review focuses on the point of view of HCWs, and finds that IMCI implementation works best with a program structure with strong leadership and governance, with HCWs speciﬁcally dedicated to scaling-up IMCI program implementation at national, governorate and district levels, with the ultimate goal of having a positive impact on child health. Unfortunately, only a few countries achieved a full scale-up and implementation frequently remains incomplete especially in PHC-facilities [40,41]. We found major barriers to HCWs’ successful delivery of IMCI at PHC-facilities, more commonly, HCWs struggled to deliver IMCI services due to non-enabling and unsupportive health systems.

The sustainability of IMCI has been considered a major concern among LMIC stakeholders since the beginning of program rollout in early 1990s. Bryce’s review [21] found that expectations of health systems improvements were only partially fulfilled based on the IMCI impact model, due to some weakness in program design execution and lack of basic health system structures. Our results parallel those of the ‘Strategic Review’ of the IMCI program conducted by the WHO and UNICEF, which reported that after 20 years, IMCI was suffering from waning interest and decreased funding, making it difficult to reach IMCI coverage at scale [42]. Our review suggests that HCWs in PHC-facilities struggle to keep up to the evolving pace in the health system, as there are major competing child health programs that make HCWs unsure of what to prioritize and how to deliver IMCI services.

The lack of training and resources, and minimal supervision after training were among the weakest areas of IMCI implementation, as identified here and in studies of other LMICs [27,32,33,43]. Our work also highlights the magnitude of the poor supervision and monitoring after the early phases of implementation of IMCI in LMICs, reflecting larger problems relating to scale-up, such as lack of political commitment, human resources issues, fragmented program management and ineffective decentralization [24,25,30,32,40,44–46]. It appears that IMCI programs are also not harmonized to other vertical programs, creating linkage issues for HCWs [25,26,47]. HCWs lack clear roles around the IMCI program and often received no clear directives from higher authorities, resulting in fragmentation of services. As a result, HCWs lost their motivation to perform their duties because of inadequate resources and mis-aligned structures. Indeed evidence from other systematic reviews and global health studies confirms that the performance, attrition and retention of frontline HCWs are linked to intrinsic and extrinsic motivational factors such as being proud of their contributions to communities, opportunities for skills improvement and self-development, supportive supervision, proper remuneration, and healthy organizational cultures and work environments [48–50]. Some recent studies, for example, highlight opportunities to motivate health care providers in terms of performance-based incentives [51,52] and team-based goals and incentives model [50].

In the light of the WHO Health Systems Framework [53], it is also clear that IMCI programs should strike a better balance between and among the six building blocks to achieve desired child health outcomes. We identified leadership and governance as a strong determinant, suggesting opportunities to proceed by refining other blocks in a flexible manner given country-specific contexts. Indeed it has already been seen that IMCI can be a lever to improve the overall existing health system [10,21,24,25]. In Egypt, IMCI was shown to strengthen the health system, improve the availability of medicines, supplies and equipment [54]. Leon, et al [55] also described how some sub-Saharan African countries (Ethiopia, Mali and Niger) used IMCI to tackle problems in the health system, especially in relation to human resources, by intensifying their delivery of basic health services through a two-tiered community health worker system. Namely, volunteers and HCWs were given clear roles and were provided with training, stipends and essential supplies to perform their work [55].

Major progress has been made in terms of HCW knowledge and child health through the accelerated implementation of IMCI when HCWs were adequately supported. Rakha, et al [54] reported that the proportion of caretakers’ knowledge on antibiotics use increased from 7% to 67% or above when the child was seen by IMCI-trained HCWs. Moreover, treatment was more often sought from community HCWs and eventually led to substantive availability of medicines as well as equipment and supplies. Masanja, et al [17] from their study in rural Tanzania, demonstrated that the IMCI program led to significant improvements in child health (such underweight, stunting, measles immunization among others) and also bolstered improvements in caretakers’ knowledge on danger signs. Mohan, et al [33] also provided evidence on the improvement of some key newborn and child care practices such as increased health seeking behavior for acute respiratory infections, and early initiation and exclusive breastfeeding.

To ensure that the IMCI program stays relevant after 20 years, further implementation research is needed to understand what works and what does not at the implementation level [56,57]. International and national efforts to strengthen district health management teams should be the linchpin of this effort, and it is imperative to update existing policies to ensure adequate fiscal space for the IMCI program at this level [18,19,58]. It is critical for the future of the IMCI program and child health that policy and implementation levels work more closely. Doherty and colleagues [58] outlined evidence supporting the presence of a well-functioning district health system as a core component in improving quality of care in child health programs, specifically the IMCI program. Egypt and Tanzania have successfully allocated a separate funding stream for IMCI within all district budgets to ensure sufficient and reliable funding of program activities [11]. The health system must also consider the importance of legitimacy and ownership at district level [58–60]. In Nepal and Democratic Republic of Congo (DRC), strengthening district health capacity has been an integral part in reaffirmation of IMCI and prevents donor-driven re-prioritization [58]. Experiences from Kenya and Tanzania also highlight that positive uptake can be attributed to empowered district leaders [61,62].

### Review strengths and limitations

Due to its precise inclusion criteria and the large scope of literature on the IMCI program, this review may have inadvertently excluded some articles discussing factors of relevance to implementation of IMCI at PHC-facility level. Although the researchers believed that the exhaustive search terms utilized in this review captured the relevant publications within the scope of interest, we might have missed other relevant studies that were not published in the databases used. However, a biomedical librarian was consulted to help finalize the search strategy, to ensure the most important articles were included. While we believe we have been able to thoroughly describe the literature on IMCI implementation challenges at the PHC-facility level, the diversity of contexts in which IMCI is implemented means that researchers should exercise caution in generalizing findings about the challenges of scaling-up of the IMCI program. Finally, while a second coder was not used to extract themes, we believe this limitation is mitigated by the involvement of co-authors sufficiently familiar with the literature to validate findings.

## Conclusion

This review demonstrates the range and extent of literature regarding conditions that impede the uptake and implementation of IMCI globally from the lens of the frontline providers. Our review highlighted opportunities to tease out some bottlenecks in program implementation, revealing four critical domains which frequently underpin a negative feedback loop. It shows that the experiences of HCWs in implementing IMCI showed demotivating realities of an unsupportive health system and weak program execution. National and international efforts are needed to magnify the adequate fiscal space for increasing investment in the IMCI program. Government leadership, along with more structured and continued resource and training support, is necessary to foster sustainable IMCI health care services within the needs of the local community. Additional evidence is required on the quality of IMCI program implementation across country contexts and study settings. In this regard, future implementation research should be conducted in PHC-facility settings delivering the IMCI to explore program processes and acceptability by HCWs.

## Supplementary Material

Supplemental Material

## Paper context

As IMCI reaches its 20-year milestone; it is critical to draw lessons from the lens of health care workers (HCWs) to amplify IMCI reach and coverage. This review provides a synthesis of HCWs key challenges in implementing the IMCI program in primary health care facilities. Further, this review provides support to the revitalization of IMCI to respond to the changing landscape in child health.
