# Supplementary material for: Key challenges of health care workers in implementing the integrated management of childhood illnesses (IMCI) program: a scoping review
Source: Glob Health Action. 2020 Mar 2;13(1):1732669. doi: 10.1080/16549716.2020.1732669 (PMC7067189; doi:10.1080/16549716.2020.1732669)
Supplement: Supplemental Material [file ZGHA_A_1732669_SM7592.docx]

**Supplemental Table 1.** Summary of Included Studies in the IMCI Scoping Review.

| TITLE, AUTHORS & YEAR | LOCATION OF THE STUDY | AIMS | STUDY DESIGN AND POPULATION | KEY CHALLENGES |
| --- | --- | --- | --- | --- |
| 1. Global implementation survey of Integrated Management of Childhood Illness (IMCI): 20 years on.  Boschi-Pinto, et al. (2018). | Multi-country (96 countries) | To assess the extent to which Integrated  Management of Childhood Illness (IMCI) has been adopted and scaled up in countries. | Quantitative design: Cross-sectional self-administered global survey.  In-country teams consisting of representatives of the ministry of health and country offices of WHO and UNICEF. | - Lack of budget for training and activities - Poor supervision and mentoring - Staff shortage and motivation - Issues on sustainability of activities |
| 2. Consistency of Integrated Management of Newborn and Childhood Illness (IMNCI) in Shire Governmental Health Institution in 2017.  Gerensea, et al. (2018). | Ethiopia | To assess the consistency and completeness of integrated management of neonatal and child hood illness in primary health care units. | Quantitative design: Institution-based Cross-sectional survey.  384 children who are under 5 years of age and treated in the health centers using IMNCI guide line. | - Quality of care issues |
| 3. Health system factors affecting implementation of integrated management of childhood illness (IMCI): qualitative insights from a South African province.  Pandya, et al. (2018). | South Africa | To explore the key determinants of IMCI delivery in a South African province, with a specific focus on health system building blocks using a health system dynamics framework. | Qualitative design.  38 health workers (from National, 9 Provincial and 52 District health structures). | - Poor perception on IMCI strategy - Lack of planning and policy guidelines - Quality of care issues - Resources constraints (essential supplies, medicines and equipment) - Staff shortage - Poor supervision and mentoring - Lack of health information system - Fragmented governance of other vertical child health programs - Leadership and Governance problems |
| 4.IMCI and ETAT integration at a primary healthcare facility in Malawi: a human factors approach.  Robertson, et al (2018). | Malawi | To understand how the programs have been integrated in a primary care setting and identify barriers and facilitators to this integration using a systems approach. | Qualitative design.  11 staff (Medical Assistants, Student Nurses and Health Surveillance Assistants) in primary health care facility. | - Staff shortage and motivation - Resources constraints (essential supplies, medicines and equipment) - Infrastructures and transport concerns - Quality of care issues |
| 5. A survey on Integrated Management of Neonatal and Childhood Illness implementation by nurses in four districts of West Arsi zone of Ethiopia.  Seid & Sendo (2018). | Ethiopia | To identify the factors compelling the execution of IMNCI by nurses in four districts of West Arsi zone of Ethiopia. | Mixed-method design: facility based cross-sectional study.  3 hospitals and 24 health centers (185 IMCI trained nurses) | - Inadequate training, mentoring and supervision - Staff shortage - Resources constraints (essential supplies, medicines and equipment) |
| 6. Adherence to the integrated management of childhood illness guidelines in Namibia, Kenya, Tanzania and Uganda: evidence from the national service provision assessment surveys.  Krüger, et al. (2017). | Namibia, Kenya, Tanzania and Uganda | To assess the adherence of health care workers to IMCI guidelines. | Quantitative design: Facility-based Cross-sectional study.  6,856 children aged 2-43 months clinically assessed by 2006 health workers. | - Quality of care issues |
| 7. Implementation of the Integrated Management of Childhood Illnesses strategy: challenges and recommendations in Botswana.  Mupara & Lubbe (2016). | Botswana | To identify the problems IMCI implementers face when tending children under 5 years. | Quantitative design: Explorative descriptive study.  32 IMCI-trained registered nurse. | - Unique features of IMCI strategy - Resources constraints (essential supplies, medicines and equipment) - Inadequate training, mentoring and supervision - Lack of budget for training - Infrastructure concerns - Quality of care issues |
| 8. ‘Better medicines for children’ within the  Integrated Management of Childhood Illness framework: a qualitative inquiry in Uganda.  Nsabagasani, et al. (2016). | Uganda | To explore health workers’ knowledge and perspectives about child-appropriate dosage formulations, their practices and experiences in the management of childhood illnesses using IMCI approach. | Qualitative design.  Two officials from the ministry of health, two district health officials and 22 health workers. | - Lack of budget for activities - Fragmented governance of other vertical child health programs - Issues on sustainability of activities - Inadequate training, mentoring and supervision |
| 9. Primary Health Care Physicians' Adherence and Attitude towards Integrated Management of Childhood Illness Guidelines in Alexandria Governorate in Egypt.  El-ayady, et al. (2015). | Egypt | To assess the degree of primary health care physicians’ adherence and attitude towards IMCI guidelines after 17 years of application. | Quantitative design: Cross-sectional descriptive study.  37 primary health care facilities (100 physician; they were observed while examining 125 children) | - Quality of care issues |
| 10. Implementation of the integrated management of childhood illness with parasitological diagnosis of malaria in rural Ghana: health worker perceptions.  Febir, et al. (2015). | Ghana | To report on perceptions of health workers on the health system factors influencing effective delivery of test-based diagnosis of malaria with IMCI. | Qualitative design.  49 health-care workers. | - Staff shortage - Unique features of IMCI strategy |
| 11. Factors influencing the implementation of integrated management of childhood illness  (IMCI) by healthcare workers at public health centers & dispensaries in Mwanza, Tanzania.  Kiplagat, et al. (2014). | Tanzania | To identify factors influencing the implementation of IMCI in the health facilities. | Mixed-method design: Cross-sectional study.  95 health-care workers. | - Resources constraints (essential supplies, medicines and equipment) - Unique features of IMCI strategy - Staff shortage - Inadequate training, mentoring and supervision - Dissatisfaction and mistrust of community |
| 12. Why don’t clinicians adhere more consistently to guidelines for the  Integrated Management of Childhood Illness (IMCI)?  Lange, et al. (2014). | Tanzania | To measure adherence to IMCI case-assessment guidelines and identify the reasons for weak adherence. | Mixed-method design.  103 trained clinicians in 82 health facilities. | - Poor perception on IMCI strategy - Staff shortage and motivation |
| 13. Use of Antibiotics within the IMCI Guidelines in  Outpatient Settings in Papua New Guinean Children: An  Observational and Effectiveness Study.  Senn, et al. (2014). | Papua New Guinea | To investigate the effectiveness and appropriateness of antibiotics prescription within the IMCI strategy. | Quantitative design: Observational and Effectiveness study.  1605 children aged 3-27 months. | - Quality of care issues |
| 14. Challenges to the implementation of the integrated management of childhood illness (IMCI) at community health centres in West Java province, Indonesia.  Titaley, et al. (2014). | Indonesia | To provide an overview of IMCI implementation at community health centers. | Quantitative design: Cross-sectional study.  148 health workers. | - Staff shortage and motivation - Resources constraints (essential supplies, medicines and equipment) - Inadequate training, mentoring and supervision - Infrastructures concerns - Dissatisfaction and mistrust of community - Unique features of IMCI strategy |
| 15. Insight into implementation of facility-based integrated management of childhood illness strategy in a rural district of Sindh, Pakistan.  Pradhan, et al. (2013). | Pakistan | To determine the factors influencing IMCI implementation at public sector primary health care facilities. | Mixed-method design.  8 stakeholders (policy makers at provincial and district level) and 8 IMCI-trained physicians. | - Poor perception on IMCI strategy - Lack of planning and policy guidelines - Leadership and Governance problems - Unique features of IMCI strategy - Inadequate training, mentoring and supervision - Infrastructures concerns |
| 16. Global challenges with scale-up of the integrated management of childhood illness strategy: results of a multi-country survey.  Goga & Muhe, (2011). | Multi-country (27 Countries) | To report the challenges to rapid IMCI case management training (ICMT) scale-up, how countries have tried to address these, and country experiences with follow-up of IMCI trainees after ICMT. | Quantitative design: Multi-country exploratory cross-sectional questionnaire survey.  27 countries. | - Lack of budget for training and activities - Leadership and Governance problems - Staff shortage - Inadequate training, mentoring and supervision - Infrastructures concerns |
| 17. Assessment of implementation of integrated management of neonatal and childhood illness in India.  Mohan, et al. (2011). | India | To assess the progress of IMNCI in India, identified the program bottlenecks, and also assess the effect on coverage of key newborn and childcare practices. | Mixed-method design.  223 districts. | - Inadequate training, monitoring and supervision - Resources constraints (essential supplies, medicines and equipment) |
| 18. Integrated management of childhood illness in Lahej, Yemen: a qualitative analysis from the perspective of health providers.  Basaleem & Amin, (2011) | Yemen | To explore the perceptions of health providers about the IMCI strategy. | Mixed-method design.  12 IMCI Trained health providers. | - Inadequate training, monitoring and supervision - Infrastructure concerns - Resources constraints (essential supplies, medicines and equipment) - Dissatisfaction and mistrust of community - Unique features of IMCI strategy |
| 19. An Evaluation of the Quality of IMCI Assessments among IMCI Trained Health Workers in South Africa  Horwood, et al (2009) | South Africa | To evaluate the performance of IMCI trained health workers. | Quantitative design: Evaluation study.  77 IMCI trained health workers in 74 primary health care clinics. | - Quality of care issues - Inadequate training, mentoring and supervision |
| 20. The rise and fall of supervision in a project designed to strengthen supervision of Integrated Management of Childhood Illness in Benin.  Rowe, et al. (2009). | Benin | To describe the frequency and quality of supervision in the trial’s intervention area and explore reasons why supervision often was not done. | Mixed-method design.  IMCI-trained health workers in the intervention area, their supervisors, and departmental managers involved with IMCI implementation. | - Leadership and Governance problems - Inadequate training, monitoring and supervision - Staff motivation - Poor perception of IMCI strategy |
| 21. Experiences of primary health care nurses in implementing integrated management of childhood illnesses strategy at selected clinics of Limpopo Province.  Vhuromu & Davhana-Maselesele, (2009) | South Africa | To explore and describe the experiences of PHCNS in implementing the IMCI strategy. | Qualitative design.  IMCI trained health workers. | - Resources constraints (essential supplies, medicines and equipment) - Staff shortage - Infrastructures concerns - Leadership and Governance problems |
| 22. Effect of the Integrated Management of Childhood Illness strategy on health care quality in Morocco.  Naimoli, et al. (2006) | Morocco | To evaluate an intervention to promote health workers’ use of the World Health Organization’s Integrated Management of Childhood Illness clinical guidelines and to identify other factors influencing quality of care received by Moroccan children. | Quantitative design: Cross-sectional study.  101 health workers in 62 facilities. | - Quality of care issues |
| 23. Programmatic pathways to child survival: results of a multi-country evaluation of Integrated Management of Childhood Illness.  Bryce, et al. (2005) | (Multi-country) Bangladesh,  Brazil,  Peru,  Tanzania, and Uganda | To compare the findings of the MCE-IMCI relative to the program expectations reflected in the IMCI impact model | Mixed-method design: multi-country evaluation study. | - Issues on sustainability of activities - Leadership and Governance problems - Lack of budget for activities |
| 24. Scaling up integrated management of childhood illness to the national level: achievements and challenges in Peru.  Huicho, et al. (2005) | Peru | To describe levels and trends in IMCI implementation. | Quantitative design: Evaluation study.  34 Districts. | - Lack of budget for training and activities - Fragmented governance of other vertical child health programs - Inadequate training, monitoring and supervision - Resources constraints (essential supplies, medicines and equipment) |
